# Endoscopic Grading and Sampling of Gastric Precancerous Lesions: A Comprehensive Literature Review

**DOI:** 10.3390/curroncol31070290

**Published:** 2024-07-05

**Authors:** Georgios Tziatzios, Dimitrios Ι. Ziogas, Paraskevas Gkolfakis, Vasilios Papadopoulos, Apostolis Papaefthymiou, Nikoletta Mathou, Athanasios Giannakopoulos, Gerasimos Gerasimatos, Konstantina D. Paraskeva, Konstantinos Triantafyllou

**Affiliations:** 1 Department of Gastroenterology, General Hospital of Nea Ionia “Konstantopoulio-Patision”, 3-5, Theodorou Konstantopoulou, 14233 Athens, Greece; pgolfakis@gmail.com (P.G.); nicolettamathou@hotmail.com (N.M.); athgian@yahoo.com (A.G.); gerasimatos.ger@gmail.com (G.G.); konparaskeva@gmail.com (K.D.P.); 2 1st Department of Internal Medicine, 251 Hellenic Air Force & VA General Hospital, 11525 Athina, Greece; dimiziog95@gmail.com; 3 Department of Gastroenterology, General University Hospital of Larissa, 41334 Larissa, Greece; vaspapadopoulos82@gmail.com (V.P.); appapaef@hotmail.com (A.P.); 4 Endoscopy Unit, Cleveland Clinic London, London SW1X 7HY, UK; 5 Hepatogastroenterology Unit, Second Department of Internal Medicine, Propaedeutic, Medical School, National and Kapodistrian University of Athens, ‘‘Attikon” University General Hospital, 77591 Athens, Greece; ktriant@med.uoa.gr

**Keywords:** gastric, precancerous, endoscopy, grading, biopsy

## Abstract

Gastric cancer remains a disease with an ominous prognosis, while early gastric cancer has a good-to-excellent prognosis, with 5-year survival rates of up to 92.6% after successful endoscopic resection. In this context, the accurate identification of patients with established gastric precancerous lesions, namely chronic atrophic gastritis and intestinal metaplasia, is the first step in a stepwise approach to minimize cancer risk. Although current guidelines advocate for the execution of random biopsies to stage the extent and severity of gastritis/intestinal metaplasia, modern biopsy protocols are still imperfect as they have limited reproducibility and are susceptible to sampling error. The advent of novel imaging-enhancing modalities, i.e., high-definition with virtual chromoendoscopy (CE), has revolutionized the inspection of gastric mucosa, leading to an endoscopy-based staging strategy for the management of these premalignant changes in the stomach. Nowadays, the incorporation of CE-targeted biopsies in everyday clinical practice offers not only the robust detection of premalignant lesions but also an improvement in quality, by reducing missed diagnoses along with mean biopsies and, thus, the procedural costs and the environmental footprint. In this review, we summarize the recent evidence regarding the endoscopic grading and sampling of gastric precancerous lesions.

## 1. Introduction

Gastric cancer (GC) currently ranks fifth among the most prevalent cancers worldwide, being the fourth leading cause of cancer-related deaths in Europe [1]. Although its incidence has been declining over the past century and several advancements are available nowadays in our therapeutic armamentarium, its 5-year survival rate remains poor, with the majority of patients diagnosed at a late stage of the disease (46–57% with stage 4 at diagnosis), limiting the treatment options [2]. Intestinal-type gastric adenocarcinoma (Lauren classification) is the most prevalent type, representing the final step in a carcinogenesis cascade which generally takes decades to progress and is inextricably related to environmental factors, mainly *Helicobaceter Pylori* (*H. pylori*) infection [3,4]. On the other hand, early gastric cancer (EGC) carries a favorable prognosis, with a 5-year survival rate ranging from 69% to 82%, given that most lesions of this type are usually amenable to endoscopic resection by techniques of endoscopic mucosal resection (EMR) or endoscopic submucosal dissection (ESD) [5]. These data underline the fact that the identification of early neoplastic lesions is of paramount importance in an effort to improve patient outcomes. Chronic atrophic gastritis (CAG) and gastric intestinal metaplasia (GIM) are deemed to be the two cardinal precancerous conditions in the stomach, particularly when they are in their extensive form, involving both the gastric antrum and body, and they are the two phenotypes considered to be high risk for giving, over time, rise to dysplasia and, eventually, the development of adenocarcinoma [6]. The guidelines by the European Society of Gastrointestinal Endoscopy (ESGE) recommend that the diagnosis and, subsequently, risk stratification of CAG and GIM should be based on a histopathology [7]. Nonetheless, the biopsy protocols may be suboptimal, as precancerous conditions are not evenly distributed throughout the stomach; hence, random samples are prone to sampling error. Moreover, endoscopists’ adherence to biopsy protocols in real-world everyday clinical practice may be poor, highlighting the need for the qualitative improvement of diagnostic upper gastrointestinal (GI) endoscopy for the detection of neoplasia [8]. A number of advanced technological endoscopic modalities incorporating CE, i.e., narrow-band imaging (NBI), flexible spectral imaging color enhancement (FICE), i-scan (i-Scan), blue-laser imaging (BLI), and magnification endoscopy, have been devised in an effort to increase the diagnostic yield for premalignant changes and EGC, with several supporting lines of evidence [9,10,11]. In this regard, an endoscopy-based strategy could be a useful alternative, allowing a thorough assessment of the gastric mucosa within an individualized approach for the early detection and surveillance of precancerous lesions among high-risk individuals [12]. This manuscript reviews the current literature, highlighting recent advances and an evidence-based approach to the endoscopic staging of gastric precancerous lesions.

## 2. Materials and Methods

A computerized search of the MEDLINE electronic database was performed for studies published up until March 2024 in the English language, applying the following key words: endoscopic grading of gastric intestinal metaplasia; prevalence AND gastric precancerous; gastric incisura AND biopsy; operative link on gastritis assessment AND gastric cancer. In order to maximize the yield, we carried out a stepwise approach, with different searches and the combination of the results at the end.

## 3. Prevalence of Gastric Precancerous Lesions

The prevalence of gastric precancerous conditions, including atrophic gastritis and intestinal metaplasia, varies across different populations and is strongly associated with fluctuations in the reported incidence of *H. pylori* infection and gastric cancer rates. Beyond geographic variations, variable patient characteristics, symptom presentation, assessment methodologies (e.g., histology, endoscopy, serology), and diagnostic criteria across prevalence studies create challenges in the estimation of the global prevalence of these gastric precursors [13]. The first systematic review assessing the worldwide prevalence of atrophic gastritis, conducted by Weck et al. [14], comprised 41 studies, with 15 based on gastroscopy and 26 on the measurement of pepsinogen concentration. The study revealed higher prevalence rates among the elderly and certain Chinese and Japanese populations (even in young patients); however, significant variations were observed across different demographic groups, further complicated by the decreased comparability of the studies due to the use of diverse diagnostic criteria. Marques-Silva et al. [15] performed a systematic review and meta-analysis of 107 studies to determine the global prevalence of chronic atrophic gastritis and intestinal metaplasia in both the general population and in patients with upper gastrointestinal symptoms. The prevalence of chronic atrophic gastritis based on biopsies was 33.4% and 31.6% among the general population and the symptomatic patients, respectively, while the occurrence rate of intestinal metaplasia was 25% and 25.4%, respectively. When considering gastric cancer incidence, an increased prevalence of both precancerous conditions was observed in high-incidence countries compared to low-to-moderate ones (41.7% vs. 22.8% for atrophic gastritis and 28.1% vs. 21.7% for intestinal metaplasia). Moreover, both precancerous conditions were 2–3 times more common in patients over 40 years of age and in those with *H. pylori* infection. Similar to the previous study, the authors reported significant heterogeneity in the patient selection criteria and diagnostic techniques throughout the involved studies. Another meta-analysis including studies conducted between 2010 and 2020 reported an overall prevalence rate of atrophic gastritis of 25% (95% CI: 18–32%). Notably, symptomatic patients were linked to a greater rate of 35% (95% CI: 21–48%) compared to asymptomatic individuals, who demonstrated a rate of 16% (95% CI: 10–22%). Furthermore, atrophic gastritis was found to be more frequent in studies using histology as opposed to serology as the diagnostic method. Additionally, *H. pylori* was confirmed as a pivotal risk factor for atrophic gastritis, with an RR of 2.40 (95% CI: 2.16–2.67) [16]. Recently, Li et al. [17] aimed to evaluate the prevalence of preneoplasmatic conditions across Asia over the last 50 years. They found an overall pooled prevalence of 26.1% (95% CI: 22.7–30.0%) for atrophic gastritis and 22.9% (95% CI: 19.7–26.6%) for intestinal metaplasia. Regarding the latter, the prevalence was higher in Eastern Asia, particularly in Japan (52.3%, 95% CI: 33.7–70.8%). Of note, the study showed a continuous decrease in the occurrence of intestinal metaplasia, from 30% between 1991 and 2000 to 21.1% between 2011 and 2022. In the meta-regression analysis, an annual decrease of −0.79% was noticed. However, this decline was not found for atrophic gastritis. Age emerged as an important factor influencing the prevalence of both conditions; nevertheless, atrophic gastritis had a notably high prevalence even among individuals aged over 30 years.

## 4. Obstacles to Implementation in Clinical Practice and How to Overcome Them

Upper GI endoscopy is advocated for as the first-line option for early gastric cancer detection [7]; however, it is an imperfect modality, as a significant proportion—up to 9.4%—of precancerous lesions may be still missed [18]. Several of either procedure- or endoscopist-related factors may contribute to this suboptimal performance, namely the presence of saliva, bubbles, or mucus, an inadequate procedure time, a lack of expertise, poor inspection of the gastric angle or posterior wall, and a greater curvature of the gastric corpus [19]. To address this issue, the ESGE provided guidance stating a number of key performance measures that should be achieved during upper GI endoscopy in order to ensure a qualitative examination [20]. Detailed guidance as well as principles and practices aiming to help physicians in their everyday clinical practice, facilitating the complete photodocumentation of the upper gastrointestinal tract, have also been recently provided within a position statement by the World Endoscopy Organization [21]. Meticulous gastric mucosal visualization is the cornerstone, and, to achieve this, a combination of air insufflation, aspiration, and mucosal cleansing techniques (simethicone) can be pursued. Upon entrance of the endoscope in the stomach, thorough rinsing with a saline solution or water should be applied, followed by the administration of mucolytic and/or defoaming agents, as they significantly improve the quality of visualization without affecting the duration of the examination, patient satisfaction, or the rate of the side effects. This could be performed either as a preprocedural small-volume *per os* ingestion or via the working channel [19,22], but not by adding simethicone to water bottles, as the guidelines recommend against this due to an increased risk for biofilm formation [23]. After the above, the adequate cleanliness of the upper GI tract can be assessed with validated scales associated with a significantly higher detection rate of clinically significant lesions (Effective Assessment of Cleanliness in Esophagogastroduodenoscopy [PEACE] system) [24]. Thereafter, adequate insufflation is another important step allowing clinicians to observe and obtain photodocumentation of the whole stomach, considering the majority of missed cancers being located in the gastric body [18]. The time dedicated to a diagnostic upper endoscopy is another significant aspect, as a clinician with an examination time longer than 7 min identifies a greater number of high-risk lesions compared to faster endoscopists [25]. Endoscopists should also be aware of traditionally difficult-to-see areas to overcome them by optimal scope manipulation.

## 5. Endoscopic Staging of Gastric Precancerous Lesions

**Figure 1:**
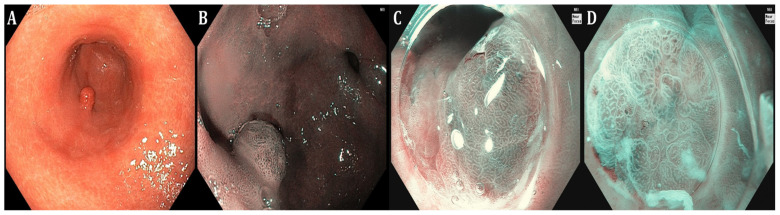
Endoscopic appearance of gastric intestinal metaplasia including marginal turbid band, light-blue crest, and white opaque substance; a hyperplastic polyp (**A**,**B**), a gastric neuroendocrine tumor (**C**), and early gastric cancer ((**D**), irregular demarcation border line, central depressed area, irregular microvascular, and microsurface pattern), using narrow-band imaging (NBI) (Olympus Medical Systems, Tokyo, Japan—photos from authors’ archive).

**Figure 2:**
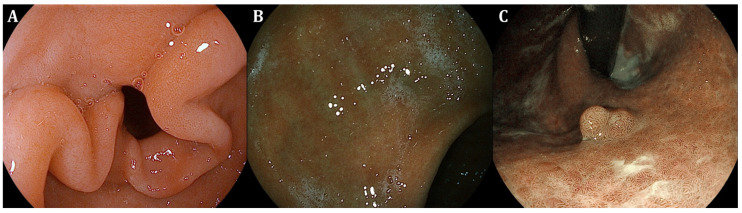
Endoscopic appearance of gastric intestinal metaplasia showing the presence of regular ridge/tubulo-villous mucosa with regular vessels in the mucosa (**A**,**B**) and a hyperplastic polyp (**C**) using i-SCAN (Pentax Endoscopy, Tokyo, Japan—photos from authors’ archive).

**Figure 3:**
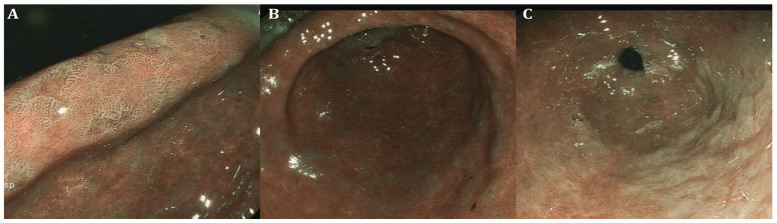
Endoscopic appearance of gastric intestinal metaplasia showing the presence of multiple light-blue crests in several areas of the mucosa (**A**–**C**) using linked-color imaging (LCI) (Fujinon, Fujifilm Medical Co., Saitama, Japan—photos from the authors’ archive).

To date, white-light gastroscopy followed by histological evaluation according to the updated Sydney System is considered the gold standard to assess GC risk in CAG and GIM, hence stratifying patients based on routine random biopsies [7]. Although available and accurate as data reports correlating with GC risk, comprehensive histopathological staging systems such as the OLGA and the OLGIM (Operative Link on Gastritis Assessment and Operative Link on Gastric Intestinal Metaplasia assessment, respectively) have not been incorporated in everyday clinical practice as they remain cumbersome. In this regard, the application of new endoscopic imaging technologies (Table 1) with virtual chromoendoscopy (Figure 1, Figure 2 and Figure 3) resulted in validated endoscopic classifications either for atrophy (Kimura–Takemoto) [26] or GIM (endoscopic grading of gastric intestinal metaplasia—EGGIM) [9] that may be also used to detect advanced stages of precancerous conditions, as cumulative evidence supports their efficacy in the real-time evaluation of GC risk based on CE visualization without the execution of routine biopsies, achieving a high correlation with the gold standard [27].

### 5.1. Kimura–Takemoto Classification for CAG

**Figure 4:**
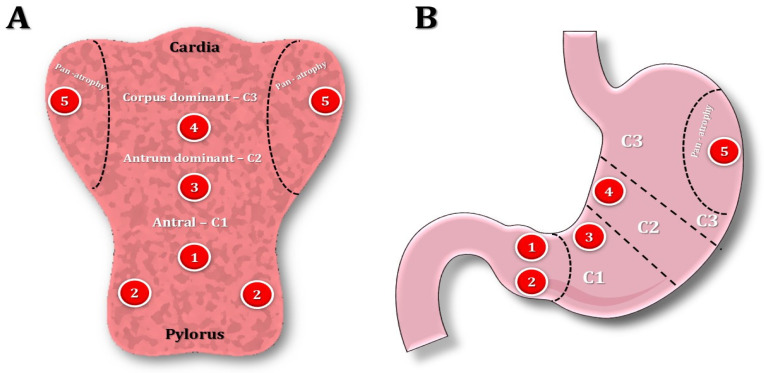
Kimura–Takemoto classification systems and updated Sydney for the endoscopic and histopathologic staging of CAG. Images depict the stomach opened along the greater curvature (**A**) and in a traditional coronal view (**B**). Endoscopic atrophy grading according to the Kimura–Takemoto classification system 60: antral (C1), antral-predominant (C2), corpus-predominant (C3), and pan-atrophy. The circled numbers refer to the location of gastric biopsies, taken according to the updated Sydney system.

The Kimura–Takemoto classification system (Figure 4) constitutes an endoscopic method to define the extent of gastric atrophy by identifying the atrophic border, which serves as the division point between non-atrophic and atrophic gastric mucosa [26]. Then, atrophy is categorized into two main types: closed and open [28]. In the closed type, the borders of atrophy remain contained within the lesser curvature, while, in the open type, the atrophy extends beyond the cardia, into the greater curvature. These primary types are further segmented into three additional subtypes: C1, characterized by atrophy limited to the antrum; C2, where atrophy affects the gastric angle or lower body; C3, where atrophy is confined to the upper area of the lesser curvature; O1, characterized by the atrophic border lying between the lesser curvature and the anterior wall; O2, defined by atrophy affecting the anterior wall of the gastric body; and O3, marked by atrophy spanning the entire stomach, resulting in the absence of folds along the greater curvature [28].

In their cross-sectional study including data of 252 patients from the United Kingdom and Japan, Kono et al. [29] demonstrated that the modified Kimura–Takemoto system achieved a favorable correlation with the histological diagnosis of gastric atrophy, with a weighted kappa value of 0.76 (95% CI: 0.71–0.80). The simplification of this system for a cancer risk-oriented classification maintained the high agreement with the histological evaluation, with a weighted kappa value of 0.81 (95% CI: 0.75–0.87). Na et al. [30] reported a prospective cohort study of 51 patients, assessing the value of an endoscopic score based on the Kimura–Takemoto classification, compared to the Operative Link for Gastritis Assessment (OLGA) system, for the evaluation of gastric atrophy. The sensitivity and specificity of an endoscopic score of 2, corresponding to the open-type Kimura–Takemoto classification, for identifying OLGA III/IV stages were 88% and 74%, respectively. Furthermore, the overall correlation coefficient between endoscopy and histology was 0.70 (95% CI: 0.52–0.81 *p* = 0.001). Few studies have evaluated the utility of the Kimura–Takemoto classification in predicting gastric cancer risk in patients with atrophic gastritis. Take et al. [31] monitored 1674 patients up to 14 years after the successful eradication of *H. pylori* and found that the incidence of gastric cancer was significantly correlated with the grade of atrophy according to the Kimura–Takemoto system before eradication (0.04% per year for mild atrophy, 0.28% per year for moderate atrophy, and 0.62% per year for severe atrophy, *p* = 0.0006). Similarly, in a retrospective study of 1823 patients, open-type atrophy was found to be an important risk factor for early gastric cancer (OR 7.19, 95% CI 2.50–20.83, *p* = 0.0003), over an average follow-up period of 63 months [32]. Song et al. [33] conducted a retrospective cohort study to identify the risk factors linked with gastric tumorigenesis among 2144 patients with gastric atrophy. The presence of gastric cancer was associated with baseline atrophy (r = 0.184, *p* < 0.001), while the C3-O1 and O2-O3 types, according to the Kimura–Takemoto classification, were considered important risk factors for tumorigenesis (HR 2.285 and HR 4.187, respectively). Moreover, in another retrospective study involving 932 *H. pylori*-positive patients (control group) and 268 patients with early gastric cancer, the frequency of O2-O3 type atrophy according to the Kimura–Takemoto grading was markedly elevated in the cancer group compared to the control group (45.1% vs. 12.7%, *p* < 0.001, respectively) [34]. A systematic review and meta-analysis summarizing data from 14 studies revealed a significantly increased risk of gastric cancer associated with both severe (O2-O3) and open-type atrophy, according to the Kimura–Takemoto classification (RR = 3.89 and 95% CI 2.92–5.17, and RR = 8.02 and 95% CI 2.39–26.88, respectively) [35].

### 5.2. Endoscopic Grading of Gastric Intestinal Metaplasia (EGGIM) Classification for GIM

The endoscopic grading of gastric intestinal metaplasia (EGGIM) was introduced as a tool for assessing GIM during an endoscopy [9]. To determine this score, image-enhanced endoscopy methods, such as NBI, are utilized, and the presence of GIM in five different regions is described and scored as 0 (no GIM), 1 (focal GIM, ≤30% of the area), or 2 points (extensive GIM, >30% of the area), providing a potential maximum of 10 points. The evaluated regions include two areas in the antrum, two in the corpus, and one in the incisura.

### 5.3. Comparison with OLGIM

Several studies have evaluated the efficacy of EGGIM through a comparison with the operative link of gastric intestinal metaplasia (OLGIM), a commonly utilized histologic grading system for GIM. In the original study by Pimentel-Nunez et al. [9], employing a cut-off score > 4, EGGIM demonstrated a sensitivity of 94.2% and a specificity of 95.2% in identifying patients with OLGIM III/IV and, thus, extensive GIM. Subsequently, Esposito et al. [36] reported on a multicenter prospective study involving 250 patients to confirm the application of EGGIM using NBI in the assessment of GIM. Overall, EGGIM demonstrated high diagnostic accuracy, and a cut-off score > 4 was the most efficient for recognizing OLGIM III/IV, with sensitivity and specificity of 89.4% (95% CI: 76.9–96.5%) and 94.6% (95% CI: 90.5–97.3%), respectively. On the contrary, 5 patients (11%) classified as OLGIM III/IV were not recognized as having extensive GIM with EGGIM, while 11 patients categorized as having no GIM according to OLGIM were diagnosed with GIM, as indicated by an EGGIM score of 1–4. In another prospective study among 37 patients, EGGIM with the blue-light imaging (BLI) endoscopy system demonstrated 100% sensitivity (95% CI: 88–100%) and 79% specificity (95% CI: 62–90%) for identifying OLGIM III–IV. Furthermore, there was a significant concordance of 84% compared to NBI in determining EGGIM intervals, suggesting that EGGIM with BLI is also reliable in assessing GIM [37]. Zhang et al. [38] performed a single-center prospective study to assess the performance of EGGIM using the linked-color imaging (LCI) system (a novel image-enhanced endoscopy technology with the ability to enhance slight differences in mucosal color) in the diagnosis of GIM. In line with previous studies, a cut-off score of >4 was determined as the most effective for the diagnosis of patients with advanced stages of GIM (OLGIM III/IV). However, it is important to note that more than half of the patients identified as having extensive GIM according to EGGIM had a lower grade of GIM, categorized as OLGIM I/II. A meta-analysis encompassing the aforementioned studies validated the effectiveness of EGGIM compared to OLGIM, with a pooled sensitivity and specificity of 0.92 (95% CI 0.86–0.96) and 0.90 (95% CI 0.88–0.93), respectively. Notably, heterogeneity was observed in the specificity rates across the selected studies, potentially due to the increased number of false-positive results reported with EGGIM in some patients [39].

### 5.4. Assessment of Gastric Cancer Risk

Besides functioning as a tool for examining the extent of GIM, few studies have explored the utility of EGGIM as a risk stratification score for gastric cancer. The first study of this aspect was a single-center, case–control study conducted in Portugal, consisting of 187 patients with early gastric neoplasia (low- and high-grade dysplasia, early gastric cancer), matched with 187 control subjects [12]. An EGGIM score of ≥5 was more common among the cancer patients compared to the control group (68.6% vs. 13.3%, *p* < 0.001). Additionally, both EGGIM scores 1–4 and 5–8 were correlated with an increased risk of gastric cancer; however, the correlation was more significant for the latter (AOR 12.9, 95%CI 1.4 to 118.6 vs. AOR 21.2, 95%CI 5.0 to 90.2, respectively). In a multicenter observational study including 380 patients (115 with known gastric cancer and 265 as the control group), a high EGGIM score (5–8) was also linked to increased gastric cancer risk [OR 1.8 (1.0–3.1)] [40]. Zheng et al. [41] conducted a case–control study comparing the efficacy of EGGIM and OLGIM in estimating the risk of early gastric cancer. Both an EGGIM score ≥ 5 and OLGIM III/IV were present more frequently among the cancer patients (58.02% vs. 12.35%, *p* < 0.001 and 56.79% vs. 7.41%, *p* < 0.001, respectively). Furthermore, in a multivariate analysis, a notable association was found between these criteria and the development of early gastric cancer (AOR: 12.33, 95% CI: 3.71–41.02 and AOR: 29.74, 95% CI: 7.49–117.94 for EGGIM ≥ 5 and OLGIM III/IV, respectively). Moreover, another prospective study performed by Kawamura et al. [42] aimed to investigate differences in both GIM evaluation and individual gastric cancer risk assessment using EGGIM, comparing magnifying and non-magnifying image-enhanced endoscopy. Utilizing both methods, a high EGGIM score (≥5) was more evident in cancer patients and was related to an escalated gastric cancer risk (OR 3.3, 95% CI, and 1.2–9.0 and OR 3.1, 95% CI, and 1.1–8.9 for non-magnifying and magnifying, respectively).

### 5.5. Gastric Cancer Risk in Autoimmune Gastritis (AIG)

In autoimmune gastritis (AIG), the decrease in parietal cells leads to chronic inflammation and atrophy, with subsequent pernicious anemia and iron deficiency as the principal clinical manifestations. Additionally, patients with AIG harbor an increased risk of developing gastric neuroendocrine tumors and adenocarcinomas, making early detection mandatory. Currently, diagnosis is achieved through histological assessment, the detection of autoantibodies, elevated serum gastrin levels, and an endoscopy. Corpus-predominant atrophy, with relative sparing of the antrum, is the main endoscopic finding in AIG, reflecting the localized inflammation in the oxyntic mucosa of the corpus [43]. Terao et al. [44] evaluated 245 cases of AIG and reported that, besides corpus atrophy, the distinctive endoscopic findings of this condition included remnants of oxyntic mucosa and sticky adherent dense mucus, occurring in approximately 30% of patients. Of note, the authors found that patchy redness and circular wrinkle-like patterns in the antrum were present in 22.1% of cases, suggesting that these findings may be indicative of AIG. However, it is important to consider that, in cases of AIG, antral inflammation could also be attributed to concomitant *H. pylori* infection or bile reflux. Recent studies have reported endoscopic manifestations of early AIG, in which inflammation occurs in the absence of mucosal atrophy. Commonly described findings include swelling of the areae gastricae, leading to a mosaic pattern, pseudopolyp-like reddish nodules, and diffuse edematous and hyperemic gastric mucosa in the corpus [43,45]. These findings need to be validated in further studies to enhance the early detection of AIG.

### 5.6. The Role of Artificial Intelligence (AI)

Recently, the implementation of artificial intelligence (AI) techniques for the early detection of gastric precancerous conditions gained significant attention. By applying various algorithms, AI technology can extract data from images and videos demonstrating gastric atrophy and intestinal metaplasia and use these data for real-time diagnosis. So far, various studies have shown that AI achieves a high diagnostic performance, at least comparable to that of endoscopists, even including experts [46,47]. In a prospective study, Zhao et al. [47] developed a diagnostic model for atrophic gastritis and found that its utilization led to a higher diagnostic rate compared to endoscopists [35.8% vs. 24.6%, χ2 = 7.962, RR = 1.453 (1.117–1.894), *p* = 0.005], including the diagnosis of moderate and severe atrophy in the gastric fundus and body. This is of paramount importance, as the detailed evaluation of these regions is usually a challenge for novice, and sometimes even experienced, endoscopists. Tao et al. [48] reported that AI correctly classified the severity of atrophy with an accuracy of 81.37% (95% CI, 73.82–88.93%), which was significantly higher than that achieved by non-expert endoscopists, suggesting that AI could also serve as an auxiliary tool for surveillance determination. A recent meta-analysis including twelve mainly retrospective studies assessed the performance of AI in recognizing intestinal metaplasia and identified pooled sensitivity and specificity of 94% (95% CI: 0.92–0.96, I^2^ = 43.71%) and 93% (95% CI: 0.89–0.95, I^2^ = 84.78%), respectively. Furthermore, AI demonstrated greater sensitivity in the diagnosis of intestinal metaplasia compared to endoscopists, without statistical significance [49]. Despite the promising results regarding the diagnostic capability of AI, it is crucial to acknowledge that the vast majority of data come from retrospective studies. It is imperative that these results be validated in more prospective studies, which better reflect daily clinical practice. Moreover, significant variation exists in the types of algorithms and the number of images used for training diagnostic models across different centers, leading to challenges in standardization and reproducibility. These pitfalls must be overcome so that AI can be widely adopted and serve as a valuable tool for the accurate diagnosis of gastric precancerous conditions [46].

## 6. Sampling of Gastric Precancerous Lesions

**Figure 5:**
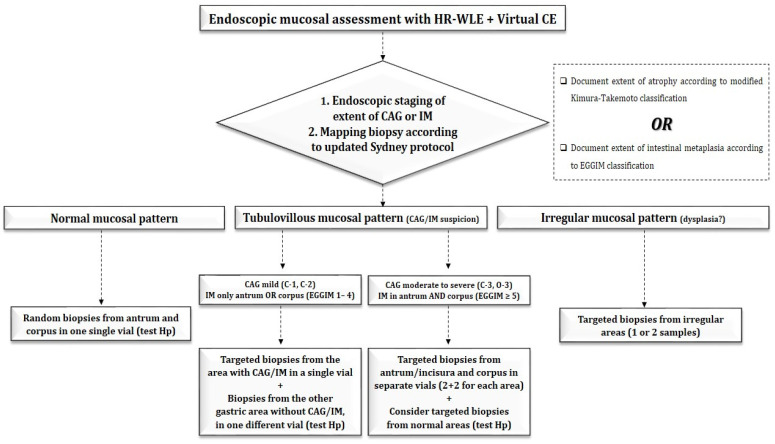
Algorithm for the assessment of the gastric mucosa for potential precancerous conditions.

Beyond any doubt, biopsy sampling holds a pivotal role in the correct staging of gastric precancerous lesions. Still, the exact number of biopsies balancing an optimal staging strategy against minimum procedural costs and a reduced environmental footprint remains to be elucidated. The current European guidelines for the management of epithelial precancerous conditions and lesions in the stomach (MAPS II) recommend performing biopsies according to the updated Sydney system: two samples from the antrum and the corpus, at a lesser and greater curvature, respectively, along the incisura angularis [7]. This notion was based on previous observations underlining the fact that the incisura is indeed the anatomical location with the highest incidence and severity of IM, and, thus, a biopsy from this site should be incorporated in biopsy protocol sampling [50,51]. Nonetheless, recent data seem to refute the role of incisura biopsy, as it offers a marginal increase in the diagnostic yield, particularly in the identification of high-risk stages (OLGA/OLGIM III/IV), posing, at the same time, a significant financial and environmental burden. In a prospective study, Verbanova et al. [52] obtained biopsies from the antrum (2), the angulus (1), and the corpus (2) from 213 patients and used them to classify the histology according to the updated Sydney system as well as the OLGA and OLGIM staging. Omitting the incisura angularis biopsy resulted in a loss of 8% of atrophic gastritis and 3% of IM (17 vs. 6/213) cases. Similar results were obtained in another recent study incorporating data from a large number (*n* = 718) of patients aiming to assess the value of adding incisura angularis biopsies to the OLGA gastritis staging system. Khomeriki and colleagues reported that the lack of the incisura angularis biopsy resulted in a decrease in the stage of atrophy only in 27 cases (7.11%), while only in 4 cases a stage downgrade from IV to III was evident, suggesting that the evaluation of histological changes in the incisura angularis, eventually, might not affect chronic gastritis staging according to the OLGA system [53]. Nonetheless, studies coming from outside of Europe seem to report contradictory results. In a Korean study investigating whether the OLGA and OLGIM scoring stages are affected by the use of different biopsy sites, the authors reported that the inclusion of an angle biopsy places significantly more patients in the high-risk group both for the OLGA and OLGIM staging (64.4% vs. 59.5%, *p* = 0.031 compared to the antrum + corpus and 48.6% vs. 37.2%, *p* < 0.001 compared to the antrum + corpus, respectively) [54]. In another observational, prospective study with 350 patients conducted in Brazil, a significant decrease in the OLGA as well as OLGIM staging systems was found when the biopsies were restricted to the corpus and the antrum (*p* = 0.008 for OLGA and *p* = 0.002 for OLGIM, respectively) [55]. A number of potential reasons explaining this discordance in the staging outcomes reported in the latter studies could be cited. First, the small size of the examined population and study design may be considered as a caveat. Second, another explanation might be speculated based on the ethnicity of the recruited population, as evidence arises from three European, two Asian, and one American studies; hence, conclusions should be interpreted under this prism. Overall, the data suggest that the addition of the incisura angularis biopsy might be pursued on a case-by-case basis when virtual CE is not available and the OLGA and OLGIM grading systems are to be used. The implementation of virtual chromoendoscopy (CE) seems to be another valuable tool, as data support that it has a higher accuracy compared to white light, minimizing the risk of missing precancerous lesions using targeted instead of random biopsies [10,27]. The CE shows a high correlation with histology regarding advanced stages of GIM, and, more importantly, it can be easily learned. Hence, the most appropriate step after index examination with white-light endoscopy is the execution of imaging-enhanced endoscopy with CE for the endoscopic staging of gastritis by guiding a biopsy for staging atrophic and metaplastic changes according to a disease-tailored, directed (targeted) strategy [56]. One of the first studies confirming this was conducted by Buxbaum and colleagues, comparing the detection of GIM with high-definition white-light (HD-WL) endoscopy, CE (narrow-band imaging—NBI), and mapping biopsies in a population with increased gastric cancer risk [57]. Among the 112 patients prospectively enrolled, NBI and mapping resulted in the detection of a higher proportion of patients with GIM compared to HD-WL [22/34 (65%) and 26/34 (76%) vs. 10/34 (29%), *p* < 0.005, respectively, for both comparisons)]. The authors came to the conclusion that HD-WL is insufficient for the detection of GIM, and using NBI-targeted biopsies plus mapping biopsies following the Sydney system would be the best approach. These results were further corroborated in a recent cross-sectional study, comparing the accuracy between targeted biopsies with the use of CE (NBI-targeted biopsy) and random biopsies (if negative for GIM read by NBI), according to the Sydney protocol [58]. The NBI-targeted biopsy achieved equivalent sensitivity (88.4% vs. 100%), specificity (90.3% vs. 90.3%), positive predictive value (88.4% vs. 89.6%), negative predictive value (90.3% vs. 100%), and accuracy (89.5% vs. 94.7%) to the Sydney protocol. The data from these studies confirm the value of targeted biopsies and point out the significance of optical diagnosis in the management of high-risk individuals for GC. A potential algorithm is displayed below (Figure 5).

### 6.1. Significance of OLGA and OLGIM Staging Systems in GC Risk Assessment

A number of individualized epidemiological studies supported an association of patients with advanced stages (III/IV) in the OLGA or OLGIM systems with an increased risk of gastric cancer. In the first Korean study, 474 patients with gastric cancer and age- and sex-matched health screening control persons were included [59]. During the multivariate analysis, OLGA stages III and IV were associated with an increased risk of gastric cancer [OR: 2.09 and *p* = 0.008 and OR 2.04 and *p* = 0.014, respectively], with this increase displaying a significant trend from OLGIM stages I to IV (OR: from 3.64 to 13.2). These results were replicated in the largest European study (*n* = 7436) with long-term follow-up (5 years) so far [60]. Considering the OLGA stage at enrollment, the rate of incident neoplasia was significantly higher for stages III and IV (17 cases, rate/103 person-years = 19.1, and 95% CI: 11.9–30.7 and 5 cases, rate/103 person-years = 41.2, and 95% CI: 17.2–99.3, respectively), underlining the fundamental role of accurate gastritis staging during endoscopy follow-up for gastric cancer. Interestingly, higher OLGA and OLGIM stages III/IV were also associated with an increased metachronous gastric cancer risk—defined as newly developed gastric cancer appearing at a new site 1 or more years after index endoscopic resection (HR 2.31, 95% CI 1.22–4.38; HR 2.36, 95% CI 1.16–4.78; and HR 2.94, 95% CI 1.34–6.95; HR 3.64, 95% CI 1.60–8.29) [61]. In the only meta-analysis available on this issue, data from eight comparative studies (six case–control studies and two cohort studies) enrolling 2700 subjects demonstrated an association for OLGA and OLGIM stages III/IV (OR 2.64, 95% CI 1.84–3.79, and I^2^ = 60% and OR 3.99, 95% CI 3.05–5.21, and I^2^ = 0%, respectively) and intestinal-type gastric cancer [62]. This conclusion seems to also apply for diffuse-type gastric cancer, as in a recent meta-analysis of six case–control and eight cohort studies both atrophic gastritis (OR = 1.9, 95% CI 1.5 to 2.4, *p* < 0.001) and intestinal metaplasia (OR = 2.3, 95% CI 1.9 to 2.9, *p* < 0.001) were associated with diffuse-type gastric cancer [63]. Moreover, this effect was found to be severity-related, as a higher atrophic gastritis/intestinal metaplasia severity correlated with a higher risk for gastric cancer (OR = 1.7, 95% CI 1.2 to 2.3, *p* = 0.002 for atrophic gastritis and OR = 1.9, 95% CI 1.3 to 2.7, *p* = 0.001 for intestinal metaplasia, respectively). Overall, this evidence shows a clear-cut association between stages III/IV of the OLGA and OLGIM classifications systems with gastric cancer risk. From the clinician’s standpoint, this could facilitate the implementation of OLGA and OLGIM grades in everyday clinical practice, which would allow specifically oriented, individualized, and highly efficient approaches for the optimal monitoring of patients with gastric precancerous lesions.

### 6.2. Impact of Reduced Biopsy Numbers on Environmental and Procedural Costs

The implementation of an endoscopy-based strategy may also have a favorable impact on less-known aspects of everyday clinical practice. Among them, the possibility of reducing the environmental footprint in the path towards sustainable GI endoscopy rises as the most compelling one [64]. The application of an endoscopy-led staging system will result, in a first step, in fewer endoscopies being performed, avoiding the environmental impact of unnecessary follow-up procedures [65]. Advanced optical diagnosis via the application and generalizability of virtual chromoendoscopy could safely reduce the number of samples sent for histological analysis and, thus, the environmental impact. Indeed, this seems to be the case, as application of the NBI-targeted biopsy strategy has been shown to lead to significantly fewer specimens compared to the Sydney protocol (311 vs. 475, *p* < 0.001), thus reducing the procedural costs and environmental impact, maintaining, at the same time, high levels of accuracy even in non-expert hands [58]. Moreover, in terms of vials, the absence of an endoscopic pattern suggestive of severe atrophy/intestinal metaplasia after meticulous mucosa inspection with CE could lead clinicians to use a single vial for biopsy specimens (for *Helicobacter pylori* diagnosis) or totally abstain from biopsies (when the status of *Helicobacter pylori* is known) [66]. To put this into practical terms, the carbon footprint of pathology biopsies is approximately 0.29 kg of CO_2_ equivalent (CO_2_e) per sample container [67]. Hence, putting three biopsies in a single vial would reduce emissions by 67%, compared to the use of three vials containing three different specimens (including the supplies, chemicals, and reagents needed for processing) [65]. In addition to this environmentally friendly aspect, evidence shows that the implementation of a CE-guided biopsy strategy during gastroscopy also reduces the medical costs in endoscopy practice. In a recent study enrolling 242 patients with GIM, the cost reduction caused by omitting biopsy and using EGGIM instead of OLGIM was examined. In their results, the authors found that EGGIM not only reduced the carbon footprint by −0.4059 kg of carbon dioxide equivalents per patient, but also led to cost savings of USD 47.36 per patient [68].

## 7. Critical Appraisal of the Evidence and Future Research Targets

While our review suggests that endoscopic scoring systems are feasible and accurate in identifying patients at a high risk for GC mainly during endoscopy, it also addresses important methodological concerns that should be taken into account and addressed in future studies. First, the Kimura–Takemoto classification for CAG and the EGGIM for GIM, respectively, are the two main endoscopic tools for the staging of gastric precancerous lesions, with a growing body of evidence in the literature supporting their use. Nonetheless, one should bear in mind that the vast majority of the studies comprising this evidence were conducted by expert endoscopists in single, tertiary referral centers, and none was of a randomized controlled design; thus, significant bias may be present, while the generalizability and interpretation of the results in terms of everyday clinical practice settings may be refutable. Although the impact of the Kimura–Takemoto classification efficacy on CAG staging is appealing, atrophy severity still lacks standardization, leading to discrepancies across the studies. Notably, some studies define O1-O3 types as indicative of severe atrophy, while others categorize O2-O3 as severe. To make things even more conflicting, most of the data derive from studies of a retrospective design, and the interaction between *H. pylori* and atrophy is not uniformly assessed. Another drawback in the existing literature is that, in terms of gastric precancerous lesions’ prevalence, there is considerable heterogeneity among the studies regarding the populations enrolled, with different baseline patient characteristics (symptoms, age), diagnosis methods (histology, serology, endoscopy), and evaluation of inconsistent endpoints. These factors contribute to variations in the observed prevalence among studies, underlining the need for establishing a consistent method. It is evident that, for GC stratification, a shift from random biopsies towards an endoscopy-based strategy with targeted biopsies is the next step. The basic prerequisite for this change, however, is for all endoscopists to be trained in the optical diagnosis of gastric precancerous conditions, so that they may not only develop but also maintain the ability and skills required to optimize the use of this technology, as the current European guidelines suggest [7]. In an effort to provide standards for optical diagnosis training, which represents the main goal for several gastrointestinal diseases, the ESGE recently published a curriculum offering guidance to endoscopists, outlining the essential steps to achieve and maintain the skill of optical diagnosis in several fields of GI endoscopy, i.e., colorectal polyps, Barrett’s esophagus, etc. [69]. Still, this is merely the first step, as training in optical diagnosis is a difficult task; it is a time-consuming process that should be performed within expert centers—two significant barriers which prevent the dissemination of knowledge about optical diagnosis to all endoscopists, reminding us that there is much ground to cover before freely recommending decisions for treatment and surveillance based on optical diagnosis standards [70].

## 8. Conclusions

The identification and accurate, robust assessment of gastric precancerous conditions is the mainstay in the early detection and successful treatment of GC. Evidence nowadays supports the notion that endoscopists should aim to perform a meticulous inspection of the entire gastric mucosa by applying a combination of high-definition WLE and enhanced-imaging modalities (NBI, iScan, FICE, and magnification) and document these findings based on validated classifications that will allow the prompt diagnosis and staging of premalignant lesions (CAG and GIM) among high-risk patients. This novel endoscopy-led staging system will be completed via targeted biopsies along with random mapping biopsies according to the Sydney protocol, maximizing our diagnostic yield and optimizing staging and GC risk assessment. This topic is very likely to grow in importance as future studies strengthen the evidence for endoscopy-based staging in different populations and settings, enabling improved accuracy and reliability and finally leading to an improved quality through an individualized diagnostic and therapeutic management model.

## Figures and Tables

**Table 1:** Image-enhanced endoscopy (IEE) modalities.

	Narrow-Band Imaging (NBI)	i-Scan	Linked-Color Imaging (LCI)
Manufacturer	Olympus Co., Tokyo, Japan	PENTAX Co., Tokyo, Japan	Fujifilm Co., Tokyo, Japan
Mode of action	Contrasting light is focused on a narrow area (30 nm) with wavelengths of 415 ± 15 nm (blue) and 540 ± 15 nm (green); differences in mucosal lesions are expressed in color and detailed images including surface mucosal blood vessels	Software-based dynamic image-enhancement technology improving surface enhancement (SE), contrast enhancement (CE), tone enhancement (TE), and tone enhancement mode g improves the visualization of grimly lit far-fi. SE emphasizes contrast based on the data obtained from each pixel and is useful for the detection of lesions. CE enhances the changes in the mucosal surface by adding a blue tint to the relatively dark area using the brightness data of the pixels. TE divides the image into red, green, and blue elements and then recombines a new image by transforming each element, emphasizing the changes.	The use of blue-violet wavelength, a short wavelength of 410 and 450 nm, in addition to the red, green, and blue wavelengths to improve mucosal abnormalities’ detection. The LCI amplifies both blue-violet- and white-light wavelengths; hence, bright red becomes more vivid, and the pale red becomes paler, improving lesion detection.
GIM endoscopic findings	Light-blue crest (LBC) as fine blue–white line on the crest of the epithelial surface, white opaque substance (lipid droplets) obscuring the subepithelial capillaries, multiple pale, elevated patches	Elevated greyish white patches surrounded by pale and normal-color gastric mucosa or blotchy patchy erythema, lipid droplets termed white opaque substance (WOS), patchy reflections of blue–white located on epithelial margins termed light-blue crest	Lavender area, “Lavender color sign” (LCS); “Purple in Mist” (purple mixed with white on the epithelium, with signs of mist detected by non-magnifying LCI observation—PIM); “patchy lavender color” (patchy lavender color with a regular mucosal pattern and a clear border—PLC)
